# Effects of leucine supplementation and resistance training on myopathy of diabetic rats

**DOI:** 10.14814/phy2.13273

**Published:** 2017-05-23

**Authors:** Carlos Eduardo C. Martins, Vanessa B. de S. Lima, Brad J. Schoenfeld, Julio Tirapegui

**Affiliations:** ^1^ Department of Food Science and Experimental Nutrition Faculty of Pharmaceutical Sciences University of São Paulo São Paulo Brazil; ^2^ Department of Health Sciences Lehman College Bronx New York

**Keywords:** Diabetes mellitus, leucine supplementation, resistance training, skeletal muscle

## Abstract

Leucine supplementation and resistance training positively influence the protein translation process and the cell signaling mTOR (mammalian target of rapamycin) pathway that regulates muscle protein balance and muscle remodeling, and thus may be therapeutic to diabetic myopathy. However, the effect of a combined intervention has not been well studied. Forty male Wistar rats were divided into five groups, control (C), diabetic control (D), diabetic + trained (DT), diabetic + L‐leucine (DL), diabetic + L‐leucine + trained (DLT). The supplementation of 5% leucine in chow, and resistance training were conducted for 8 weeks postweaning of rats. The extensor digitorum longus was used to assess signaling proteins involved in muscle protein synthesis, and the gastrocnemius and soleus were used for determination of muscle weight. Blood samples were collected for biochemical assays. Strength and ambulation tests were employed to evaluate motor performance. Results showed that both leucine supplementation and resistance training elevated the activity of mTOR‐p70S6K in diabetic rats (*P* < 0.05). Moreover, though leucine supplementation in combination with resistance training demonstrated synergistic effects on p70S6K (*P* < 0.05), both treatments were capable of recovering motor performance (*P* < *0.05*). In conclusion, 5% leucine supplementation combined with resistance training has the potential to attenuate muscle loss and motor performance decrements in diabetic rats, at least in part through increased protein synthesis.

## Introduction

Diabetes mellitus is a chronic inflammatory disease that affects nearly every biological process including an altered protein metabolism, decreased muscle mass, and in some cases, impaired ability to carry out daily activities, decreased productivity and diminished quality of life (Workeneh and Bajaj 2013). While the development of each form of diabetes (type 1 or type 2) drastically differs, resultant pathologies often overlap. In each diabetic condition, a failure to maintain healthy muscle is often observed, and this is termed diabetic myopathy (D'Souza et al. 2013; Krause et al. 2013).

Attenuating the effects of diabetes can be challenging, but recent studies provide important insights into the cellular and molecular etiology of diabetes‐associated decreases in muscle mass, and these findings may be useful in the development of new therapies for the disease (Gumucio and Mendias 2013). In addition, studies have shown that leucine supplementation and resistance training confer an anabolic effect by stimulating the beginning of the protein translation process and the cell signaling pathway known as mTOR (mammalian target of rapamycin), thereby promoting positive effects on strength gains, muscle mass, protein balance, and preservation of functional capacity (Hurley and Roth 2000; Castaneda et al. 2002; Dreyer et al. 2006).

Resistance training induces an increase in muscle protein synthesis (MPS) that can last for 48 h postexercise (Phillips et al. 1997) and the repetitive strain of regimented weekly sessions promotes a positive protein balance that can lead to substantial accretion of muscle proteins over time. A positive protein balance requires that the rate of MPS exceeds the rate of muscle protein breakdown (MPB) (Burd et al. 2009). Hypertrophy of skeletal muscle and the resulting concomitant gain in strength are of great interest for people with disease‐induced atrophy. There is evidence that resistance training can stimulate and reinforce cellular and molecular processes that lead to a compensatory hypertrophy response in this population (Haddad and Adams 2002). Moreover, resistance training has been shown to augment insulin signaling and glucose transport in skeletal muscle tissue (Takala et al. 1999; Holloszy 2005).

The intake of dietary protein after resistance training increases plasma amino acids, which results in the activation of signaling molecules leading to increased MPS and muscle hypertrophy (Phillips 2014). Although essential amino acids are necessary for hypertrophy, L‐leucine has been specifically shown to activate mTOR and increase the initiation of the translation of protein synthesis (Guimarães‐ferreira et al. 2014). On the other hand, the hypothesized beneficial effects of leucine supplementation for the treatment of diabetes mellitus are related to the improvement of energy balance, net protein balance, and glucose homeostasis (Pedroso et al. 2015).

In order to better elucidate the actions of leucine supplementation and resistance training (alone or combined) on muscle remodeling processes, the purpose of this study was to investigate the chronic effects of these interventions on muscle atrophy in an experimental model of diabetes mellitus induced by streptozotocin (STZ).

## Materials and methods

### Animals

Five‐day‐old male Wistar Hannover rats were separated from their mothers for 8 h. They were divided into two groups: the diabetic group (D group) received an ip injection of STZ [120 mg/kg body weight (b.w.)] freshly diluted in citrate buffer [10 mmol/L Na citrate (pH 4.5)]; and the nondiabetic group (non‐D group) received only the vehicle (ip) in an equivalent volume and served as control [control group (C group)] (*n = *8*)*. After weaning, rats from the D group were divided into four groups: D group (D, *n = 8*) received an isonitrogenous control diet; DT group (DT, *n = 8)* received an isonitrogenous control diet and performed resistance training; DL group (DL, *n = 8*) was treated with 5% L‐leucine; and DLT group (DLT, *n = 8*) was treated with 5% of L‐leucine and resistance training. After weaning, the animals presenting glycemia above 150 mg/dl were included in this study.

The animals were obtained from the Animal House of the Faculty of Pharmaceutical Sciences, University of Sao Paulo and the experiments were performed according to the ethical guidelines adopted by the Brazilian College of Animal Experimentation (protocol 451). The animals were maintained in individual cages under standard conditions of light (12‐h light/dark cycle), temperature (22 ± 2°C) and relative humidity (55 ± 10%), and with full access to food (American Institute of Nutrition) and water. The body weight, food intake and water intake were registered three times per week from weaning to killing (12‐week‐old). All animals were adapted to the laboratory environment for 1 week, before engaging in the training program. Animals were euthanized under tiletamine‐zolazepam and fentanyl anesthesia at 48 h after the last resistance training session and with a 6‐h fast. The extensor digitorum longus muscle (EDL), gastrocnemius (GAS), soleus (SOL) muscles of each limb were isolated, weighed, and frozen at −80°C. The protein concentration in EDL muscle homogenate was determined with a BCA protein assay kit (Thermo Scientific, Rockford, IL, USA).

### Diets

The experimental diets were prepared according to the recommendations of the American Institute of Nutrition (Reeves et al. 1993). After weight distribution, rats were fed either the control diet (C group), an isonitrogenous control diet (D and DT group), or a leucine‐rich diet (DL and DLT group) for 8 weeks. 5% of L‐leucine was added to the intervention diet by replacing equal amounts of corn flour, while the isonitrogenous control diet contained an additional 4% of nonessential amino acids (Table 1). The nonessential amino acids meal was used as the control diet to standardize the nitrogen (Vianna et al. 2012). The supplemental dose of 5% L‐leucine was used because it has been shown in previous work to positively regulate MPS (Rieu et al. 2003). Other research used this leucine dose with no toxic effects reported (Tsubuku et al. 2004).

**Table 1:** Diet composition^a^ (%)

Ingredients	Basal diet	Leucine‐rich diet	Isonitrogenous control diet
Cornstarch	62%	57%	58%
Casein	14%	14%	14%
Sucrose	10%	10%	10%
Soybean oil	4%	4%	4%
Cellulose	5%	5%	5%
Mineral mixture	3%	3%	3%
Vitamin mixture	1%	1%	1%
L‐cystine	1%	1%	1%
Choline bitartrate	0.2%	0.2%	0.2%
Tert‐butyl hydroquinone	0.0008%	0.0008%	0.0008%
Leucine	0%	5%	0%
NEAAs	0%	0%	4%

NEAAs‐ isonitrogenous mixture of nonessential amino acids (alanine, aspartate, glutamate, glycine, proline, and serine).

a Based on AIN‐93M (Reeves et al. 1993).

### Resistance training protocol

The resistance training protocol was adapted from Hornberger and Farrar (2004), which consisted of climbing a vertical ladder (1.1 × 0.18 m, 2 cm grid, 80° inclined) with progressive loads secured to the base of the rats tail, as described below. The load apparatus was secured to the tail by wrapping the proximal portion of the tail with a self‐adhesive foam strip. A Velcro strap was wrapped around the foam strip and fastened. With the load apparatus secured to the tail, the rats were placed at the bottom of the ladder and familiarized with climbing. The initial climb consisted of carrying a load that was 75% of the animal's body mass. After this, an additional 30 g weight was added until a load was reached with which the rat could not climb the entire length of the ladder. Failure was determined when the animal could not progress up the ladder after three successive stimuli to the tail. The highest load successfully carried the entire length of the ladder was considered the rat's maximal carrying capacity for that training session. Training sessions were 9 ladder climbs with 75%, 90%, and 100% of the rat's previous maximal carrying capacity, determined earlier. During subsequent ladder climbs, an additional 30 g load was added until a new maximal carrying capacity was determined.

### Motor performance tests functional assessment

In order to evaluate skeletal muscle function, the animals were allowed to grab onto the Grip Strength System (model: DFE‐002, San Diego Instruments, San Diego, Cal, USA) with their forepaws as the experimenter gently pulled on their tails. The result is the maximal force before the animal releases the forepaws of the bar (mean of three measurements of maximum pull) (Anderson et al. 2004; Nalbandian et al. 2013).

The second motor performance test was the ambulation test, which was performed to determine the mean length and width (cm) of a step measured in hind foot ink prints while mice freely ran along a corridor (length: 100 cm; width: 10.5 cm; height of lateral walls: 20 cm) three different times. Before the test, the animals were permitted to explore the apparatus (Kennel et al. 1996; Zanchi et al. 2012). Mean values were individually calculated for each test through the mean of three consecutive tests performed.

### Serum glucose and insulin levels

Blood was collected and centrifuged in order to obtain the serum fractions. Serum was stored at −80°C for further analysis. Serum glucose was measured using commercial available kits (Labtest^®^). Serum insulin levels were measured using commercial RECYTMAG‐65k MILLIPLEX MAP rat cytoquine/chemoquine magnetic bead.

### Western blot analysis

Initially, frozen EDL muscle in ice‐cold lysis buffer: 50 mmol/L phosphate buffer (pH 7.0), 0.3 mol/L sucrose, 0.5 mmol/L dithiothreitol, 1 mmol/L EDTA, 0.3 mmol/L PMSF, 10 mmol/L NaF, 1:100 Phosphatase Inhibitor Cocktail 1–2 (Sigma‐Aldrich, St. Louis, MO) and 1:100 Protease Inhibitor Cocktail (Sigma‐Aldrich) using a polytron homogenizer. After homogenization and transfer to new prechilled tubes, samples were centrifuged at 13,000*g* for 25 min at 4°C. Supernatants were collected and total protein concentrations were measured using a BCA protein assay kit (Pierce, Rockford, IL). Lysates were combined with a sample buffer: 240 mmol/L Tris (pH 6.8), 40% glycerol, 0.8% sodium dodecyl sulfate (SDS), 200 mmol/L beta‐mercaptoethanol, and 0.02% bromophenol blue. Proteins (25 *μ*g) were subjected to SDS polyacrylamide gel electrophoresis and transferred onto nitrocellulose membrane. Membranes were blocked with 5% bovine serum albumin (BSA) and phosphate buffered saline with tween (PBST) and were incubated overnight at 4°C with rabbit polyclonal antibodies (Cell Signaling Technology, Beverly, MA) diluted in PBST buffer: anti‐mTOR diluted 1:1000, anti‐pmTOR (Ser2448) diluted 1:1000, anti‐S6K1 diluted 1:1000, anti‐pS6K1 (Thr389) diluted 1:1000. Membranes were incubated for 1 h with horseradish‐peroxidase‐labeled antirabbit IgG antibodies (Sigma‐Aldrich) diluted 1:1000 in PBST buffer and 1% BSA. Blots were visualized for 5 min in an ImageQuant 400 (GE Healthcare, Amersham, UK) image system with a solution prepared with ECL‐Advance Western blotting Reagent (GE Healthcare). After densitometry, phosphorylated proteins were normalized by total protein content. A horseradish‐peroxidase‐labeled anti‐*β*‐actin (Sigma‐Aldrich) diluted 1:40,000 was used in order to verify correct protein loading.

### Statistical analysis

Statistical analysis was performed using GraphPad Prism software 6.0 (GraphPad Software, Inc., San Diego, CA). 2‐way ANOVA test with repeated measures followed by Tukey's test was used for comparison of weekly evolution of body weight and (time × group) of functional performance tests, and 1‐way ANOVA followed by Tukey's test for food and water intake, muscle weight, glucose and insulin and western blot analysis. The a priori significance level was set at *P* < 0.05. The results are expressed as means ± SEM.

## Results

### Body weight

**Figure 1:**
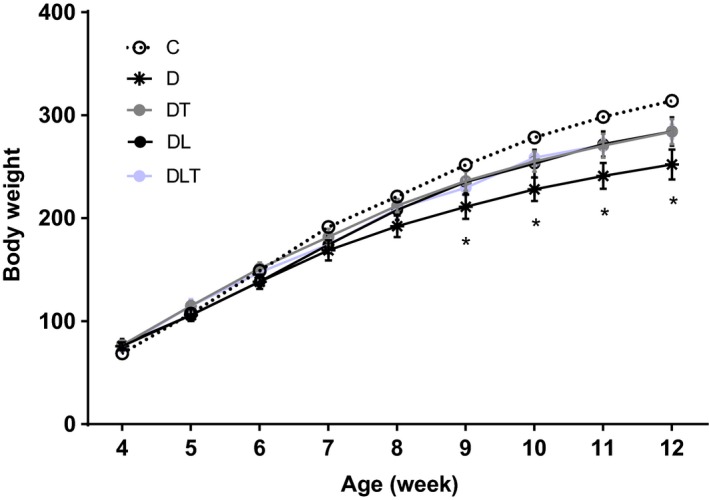
Changes in body weight between eight time‐points within each group during the 8 weeks of the experiment (*n* = 8 per group). C: nondiabetic control group; D: diabetic group; DT: resistance training group; DL: leucine supplementation group; DLT: leucine supplementation plus resistance training group. *P <* *0.05 C group vs. D group.

The body weight of nontreated diabetic rats (D group) was reduced in final time course of 8 weeks when compared with nondiabetic rats (C group) (*P* < 0.05). Interestingly, the body weight of groups (DT, DL and DLT) that received treatment was not significantly different when compared with group C (*P* > 0.05) (Fig. 1).

### Food and water intake

In nontreated diabetic rats (D group) food intake was significantly (*P* < 0.05) enhanced as compared with control rats (C group). Both groups (DT, DL and DLT) had lower food intake when compared to diabetic rats (D group). However, only food intake was normalized in diabetic rats with leucine supplementation (DL group) (Table 2). In terms of water intake, the water intake of the D group was significantly (*P* < 0.05) enhanced as compared with C group, DT group, DL group, and DLT group. No statistical differences (*P* > 0.05) were noted in relation to the other groups (Table 2).

**Table 2:** Characteristics of the male rats

Groups	C	D	DT	DL	DLT
EDL (g)	0.137 ± 0.002	0.106 ± 0.007^a^	0.115 ± 0.002^a^	0.114 ± 0.003^a^	0.114 ± 0.002^a^
EDL (g/100 g)	0.040 ± 0.000	0.039 ± 0.002	0.040 ± 0.001	0.041 ± 0.001	0.043 ± 0.000
GAS (g)	1.591 ± 0.027	1.365 ± 0.072^a^	1.565 ± 0.54	1.502 ± 0.047	1.502 ± 0.047
GAS (g/100 g)	0.470 ± 0.007	0.467 ± 0.009	0.545 ± 0.007^a^, ^b^	0.500 ± 0.007^c^	0.570 ± 0.024^a^, ^b^
SOL (g)	0.116 ± 0.004	0.101 ± 0.005	0.116 ± 0.002	0.112 ± 0.004	0.112 ± 0.007
SOL (g/100 g)	0.035 ± 0.000	0.085 ± 0.046	0.0417 ± 0.002	0.037 ± 0.000	0.040 ± 0.000
Glucose (mg/dl)	85.89 ± 7.29	310.8 ± 57.38^a^	175.5 ± 10.18^b^	181.8 ± 21.11^b^	176.5 ± 27.78^b^
Insulin (pg/ml)	3022 ± 406	1362 ± 282^a^	2183 ± 576	1622 ± 399	1911 ± 320
Food Intake (g/24 h)	15.1 ± 5.27	42.8 ± 5.91^a^	30.1 ± 2.73^a^, ^b^	17.9 ± 6.04^b^	22.6 ± 2.27^a^, ^b^
Water Intake (ml/24 h)	33.2 ± 3.28	85.14 ± 27.54^a^	41.65 ± 5.11^b^	26.21 ± 1.96^b^	27.62 ± 1.98^b^
EDL Protein (mg/g body weight)	0.0910 ± 0.002	0.0639 ± 0.006^a^	0.0967 ± 0.003^b^	0.0773 ± 0.000	0.1041 ± 0.011^b^

Muscle weights in g and g/100 g. Glucose and insulin represents at the last week post euthanasia measured in 6‐h‐fasted. Food and water intake for the last week of experiment. C: nondiabetic control group; D: diabetic group; DT: resistance training group; DL: leucine supplementation group; DLT: leucine supplementation plus resistance training group (*n* = 8 per group).

a Vs. C Group (*P *<* *0.05).

b Vs. D Group (*P *<* *0.05).

c Vs. DLT Group (*P *<* *0.05).

### Muscle weight

After the 8 week experimental period, the absolute weight of the EDL and GAS muscles were lower in the D group compared to the C group (*P* < 0.05), indicating muscular atrophy in this experimental model. Supplementation with leucine and resistance training did not change the absolute and relative weight of the EDL muscle of diabetic animals. However, there was no statistical difference in the absolute weight of the GAS muscle in the DL, DT, DLT groups compared with C group (*P* > 0.05), suggesting that leucine and/or resistance training may attenuate the muscle mass loss detected in untreated diabetic animals. Moreover, the relative weight of the GAS (absolute muscle weight/100 g of body weight) of the DT and DLT groups was higher compared to the D group, and was higher that the C group, demonstrating the anabolic effect of resistance training (*P* < 0.05). There was no statistical difference in the absolute and relative weights of SOL between the experimental groups (*P *>* *0.05), in the absolute and relative weights of SOL between the experimental groups (*P *>* *0.05), and between the DL and D groups in the relative weight of the assessed muscles; in other words, chronic supplementation of leucine did not affect this parameter in diabetic rats (Table 2).

### Glucose and insulin on serum

The D group had higher fasting glucose compared to other groups, with DT, DL, and DLT showing significant improvements in this outcome (*P* < 0.05). With respect to serum insulin levels, the D group presented impaired fasting insulin compared with nondiabetic rats (*P* < 0.05). Supplementation of leucine and resistance training improved fasting insulin (*P* < 0.05). Although no statistical difference was noted between the diabetic animals, the DT, DL and DLT groups modestly increased insulin compared to the D group. These data are shown in Table 2.

### Total protein in EDL muscle concentration

Muscle protein content is reported as mg/g of body weight (Adams and Haddad 1996). The muscle protein content in C, DT, and DLT groups were significantly greater as compared with D (*P* < 0.05), but no difference was observed between DT and DLT.

### Functional performance tests

**Figure 2:**
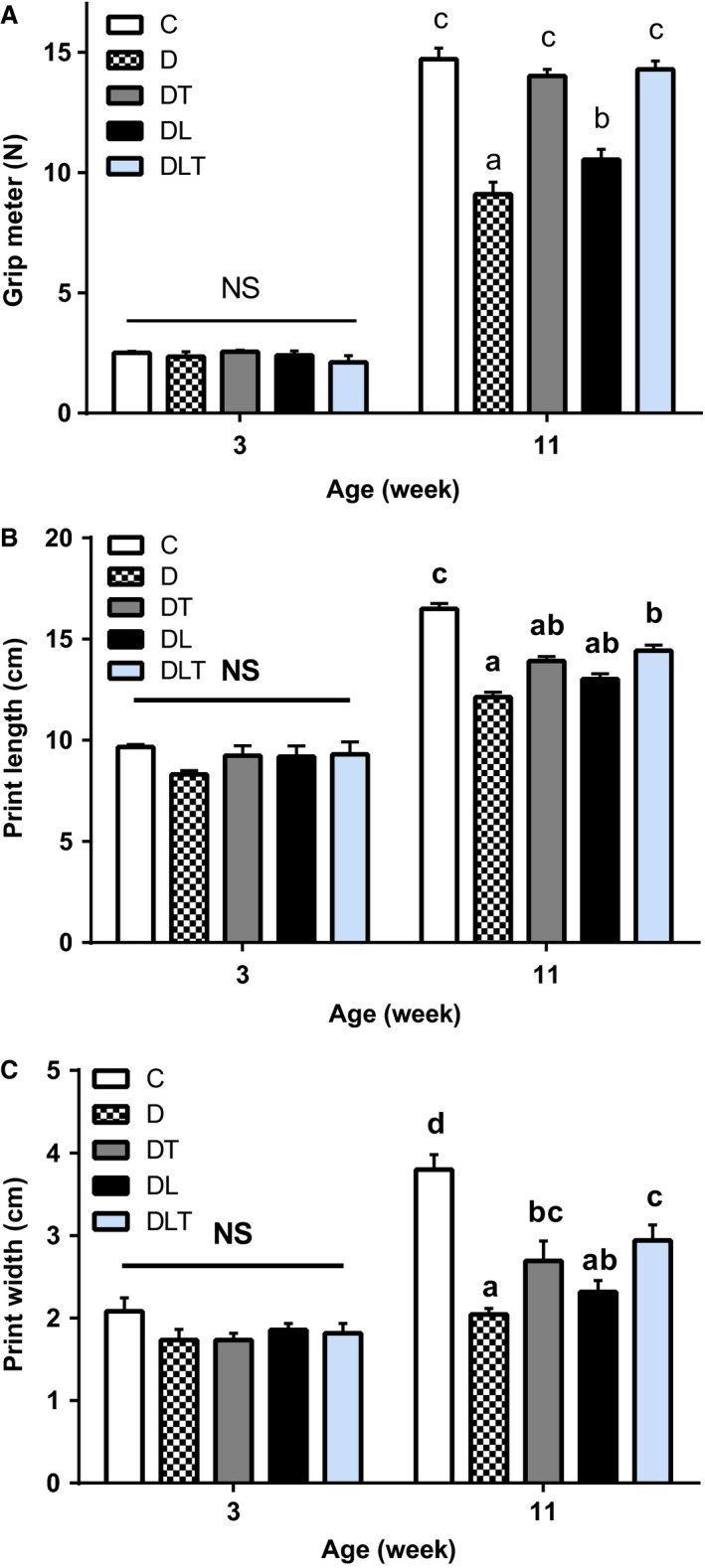
Functional performance tests. (A) grip meter (N); (B) ambulation (length in cm); (C) ambulation (width in cm). C: nondiabetic control group; D: diabetic group; DT: resistance training group; DL: leucine supplementation group; DLT: leucine supplementation plus resistance training group. *N* = 8 per group. Means with different letters are significantly different (*P *<* *0.05).

To evaluate the effects of training and leucine supplementation on muscle performance, grip strength and ambulation tests were administered. Results for these data are presented in Figure 2. Resistance training (alone or combined) showed statistically greater functional improvements when compared to the D group (grip strength – Fig. 2A, *P* < 0.05 and footprint length and width – Fig. 2B and C, respectively, *P* < 0.05). These results suggest that leucine supplementation alone was able to significantly mitigate the loss of strength in diabetic rats, but resistance training was superior for promoting gains in muscle strength in comparison with leucine‐supplemented animals, as shown in Figure 2A (*P *<* *0.05). With respect to the 1‐repetition maximum (1RM) on the amparate (ladder), the DL group increased strength by 12% increase while the DLT and DT groups showed increases of 102% and 101%, respectively, compared to the D group (data not shown). In the ambulation test, DLT was the only group in which there was attenuation of the motor function worsening in terms of length between its steps when compared to group D. The DT and DLT groups had greater width between their steps when compared to the group D, and this parameter was superior in DLT compared with DL (*P *<* *0.05). Once again, these results indicate that resistance training was fundamental for improvement of physical and motor function in diabetic animals.

### Protein expression with relation to muscle protein synthesis

**Figure 3:**
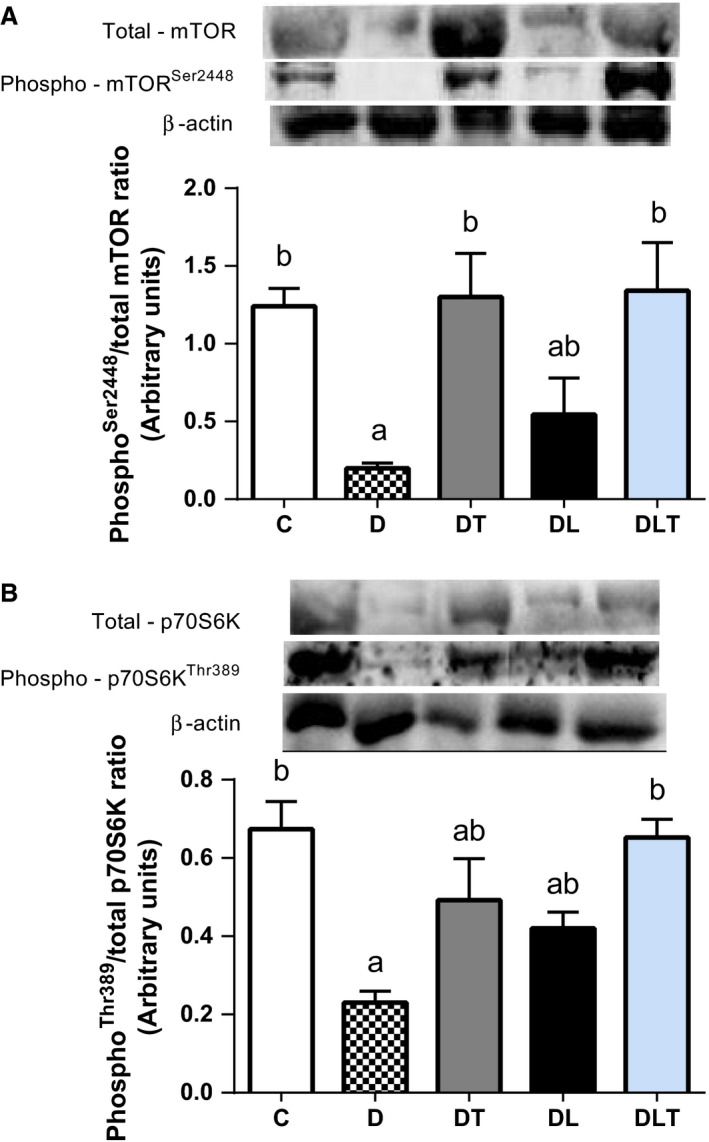
Effects of leucine supplementation and resistance training on protein expression with relation to muscle protein synthesis. (A) Representative blots show the effect of leucine and resistance training on the EDL muscle phospho/total ratio of mTOR, (B) phospho/total ratio of p70S6K. C: nondiabetic control group; D: diabetic group; DT: resistance training group; DL: leucine supplementation group; DLT: leucine supplementation plus resistance training group. *N* = 8 per group. Means with different letters are significantly different (*P *<* *0.05).

To elucidate potential mechanisms responsible for the anabolic effect of leucine and resistance training on STZ‐induced muscle atrophy, we measured mTOR and p70S6K protein levels in EDL muscles. In nontreated diabetic rats phospho^Ser2448^/total mTOR ratio and phospho^Thr389^/total p70S6K ratio protein was reduced when compared to the control group (C group) and the DT and DLT groups (*P *<* *0.05). Therefore, resistance training enhanced recovery of mTOR and p70S6K proteins (*P *<* *0.05), but leucine alone did not affect these parameters (Fig. 3A and B).

## Discussion

This study investigated the effects of chronic supplementation with leucine and resistance training on diabetic myopathy in experimental diabetes mellitus induced by streptozotocin. Our results showed that resistance training alone or combined with leucine minimized the deleterious effects of diabetes mellitus on skeletal muscle as determined by an enhanced physical functional capacity muscle as well as improvements in certain metabolic parameters such as fasting glucose and insulin. These results appear to be mediated by an increased activation of the molecular signaling pathways involved in MPS.

In diabetes mellitus, muscle atrophy associated with diabetic myopathy may be the result of reduced MPS and/ or exacerbated increase in muscle protein breakdown. It is known that skeletal muscle is approximately 40% of the total body mass (Frontera and Ochala 2015), thus, any degenerative disorder leading to muscle atrophy is associated with a decrease in quality of life along with an increased risk of morbidity and mortality (Goodman et al. 2011; Srikanthan and Karlamangla 2014).

The results of this study show that combined resistance training with leucine supplementation recovered the activity of the mTOR‐p70S6K pathway in diabetic animals, which may reflect greater MPS. This suggests that the anabolic stimulation of resistance training is the predominant factor in the activation of this pathway in diabetic animals, and leucine may enhance this effect as a substrate for muscle protein translation. Evidence shows that leucine directly activates mTOR after a resistance training bout. Pasiakos et al. (2011) demonstrated that leucine supplementation after exercise increased MPS by 33% in conjunction with heightened intracellular signaling. Similarly, Dreyer et al. (2008) found that a leucine‐enriched supplement provided after resistance training increased both mTOR signaling and mixed muscle protein synthesis.

Resistance training induces muscle hypertrophy via a sequential cascade consisting of: (1) activation of muscle fibers; (2) signaling pathway activation resulting from the mechanical deformation of the muscle fibers; (3) release of hormones and myokines involved in the immune/inflammatory response; (4) an increased MPS from the activation of protein transcription and translation, (5) an increase in the cross‐sectional area of muscle fibers (Baar and Esser 1999; Kubica et al. 2005; Spiering et al. 2008; Ogasawara et al. 2013). The load‐induced activation of mTOR has been shown to be a critical component in this hypertrophic cascade (Burd et al. 2010; Goodman et al. 2011). Thus, the influence of resistance training on the activation of the mTOR‐p70S6K pathway would appear to explain functional improvements in diabetic animals that performed resistance training.

In our study, supplementation of leucine (without mechanical stimulation of resistance training) did not significantly affect the mTOR‐p70S6K pathway. It is possible that the duration of treatment or the amount of leucine provided in the diet was lower than necessary to overcome muscle anabolic resistance in diabetes. Leucine stands out among the essential amino acids for its ability to stimulate protein translation and subsequently increase the MPS rate and muscle growth (Norton and Layman 2006; Kelleher et al. 2013). However, the dosages of leucine must be sufficiently high to promote optimal anabolic effects and thus mediate the synthesis of myofibrillar proteins, which are impaired in DM (Manders et al. 2012). In diabetes, ketoacidosis increases the branched chain amino acid oxidation rate by the branched‐chain *α*‐ketoacid dehydrogenase enzyme, particularly leucine, and the inflammatory state increases protein degradation rates (Workeneh and Bajaj 2013). This phenomenon may have hindered the effect of leucine on the activation of the mTOR ‐p70S6K in diabetic animals.

The activation of mTOR‐p70S6K anabolic pathway through resistance training combined with a diet supplemented with leucine was crucial to attenuate the skeletal muscle atrophy in diabetic rats. Our findings showed no statistical difference in the absolute weight of the gastrocnemius muscle of the DL, DT and DLT groups compared with the C group, suggesting that leucine and resistance training can alleviate the muscle mass loss detected in untreated diabetic animals. The relative weight of the gastrocnemius in the groups that underwent strength training (DT and DLT) increased approximately 16% and 22%, respectively, compared to the diabetic rats without treatment (D group), which highlights the anabolic effect of resistance training on the increase in muscle mass. Although results were not statistically significant, the relative weight of the gastrocnemius muscle in the group that only received leucine supplementation increased by 7% compared with the D group. These data suggest that in diabetic animals, the anabolic effects of leucine depend on the mechanical stimulus of resistance training, unlike what occurs in healthy subjects (Zanchi et al. 2012).

Supplementation with leucine and resistance training (alone or combined) did not alter the absolute and relative weights of the EDL muscle and body mass. It is conceivable that the null findings may be attributed to the short study period (8 weeks); a small muscle such as the EDL may require a longer intervention time for significant muscle hypertrophy to be observed. On the other hand, protein concentrations of EDL in C, DT and DLT rats were significantly increased compared with D group. These changes may further suggest that resistance training promotes protein accretion in EDL muscle, without affecting variations in skeletal muscle.

With respect to functional and motor elements, resistance training alone or combined with leucine supplementation recovered muscle strength and significantly improved ambulation capacity (length and width between steps) in the diabetic animals. Our study showed that grip strength was greater in DT and DLT groups compared with the D group, with no statistical difference between the trained groups and the C group, that is, the resistance training increased muscle strength in diabetic rats, even in absence of leucine. Similar findings were reported in studies of Call et al. (2010) and Chen et al. (2014). On the other hand, Kim et al. (2015) found that resistance training did not improve grip strength in diabetic Zucker rats. The reasons for the discrepancies between studies are not clear.

Interestingly, we observed that supplementation with leucine alone was able to attenuate the loss of muscle strength of diabetic animals. However, the change in grip strength of the DT group was greater than that of the DL group, demonstrating that resistance training is the primary stimulus for promoting improvements in this parameter. Contrary to our findings, Zanchi et al. (2012) showed that a diet supplemented with leucine impaired motor performance in mice with muscular atrophy induced by dexamethasone.

Supplementation of leucine and resistance training improved glycemia in diabetic animals. In our study, the treated diabetic animals (DT, DL, and DLT) showed lower fasting and postprandial values compared to the D group, with no statistical difference with the C group. The findings demonstrate that these interventions were able to normalize blood glucose levels in our experimental diabetes model. Interestingly, supplementation of leucine alone induced this effect on blood glucose levels in diabetic animals, similar to results found by Wilkinson et al. (2013) in humans with type 2 diabetes. Moreover, when administered in high doses, leucine supplementation may impair insulin signaling and the uptake of glucose in obese rats fed with a high‐fat diet, as shown by Ham et al. (2014). Due to the controversies over the role of leucine in glucose homeostasis, more studies involving this approach are needed to improve our understanding of the topic.

In this study, untreated diabetic animals displayed moderate hypoinsulinemia that is characteristic of diabetes in the experimental model used. Chronic supplementation of leucine and resistance training did not reverse hypoinsulinemia in diabetic animals, but it was able to improve the concentration of serum insulin in these animals. Some studies have shown that the resistance training is able to increase insulin production and secretion in isolated pancreatic islets of rats with uncompensated type 1 diabetes (Oliveira et al. 2010; Huang et al. 2011). Moreover, resistance training increases insulin sensitivity in peripheral tissues such as skeletal muscle and glucose uptake, with these effects persisting for up to 24–48 h after an exercise session (Holten et al. 2004; Cartee 2015).

Interestingly, we observed that supplementation of leucine alone was able to improve the serum insulin in diabetic animals. This may be explained by the effects of leucine as an insulin secretagogue, which occurs through two potential mechanisms: leucine undergoes deamination to then produce alpha‐ketoisocaproate (Gao et al. 2003) or it allosterically activates the enzyme glutamate dehydrogenase (Smith and Stanley 2008). Both mechanisms increase the intracellular ATP/ADP ratio and cause the release of insulin (rapid phase secretion of this hormone). This improvement in the concentration of serum insulin in diabetic animals after chronic supplementation of leucine may also have contributed to these animals presenting lower blood sugar levels.

In addition, leucine supplementation as well as resistance training was able to reduce clinical symptoms such as polyphagia and polydipsia present in diabetes. Studies in animals and humans suggest that leucine acts as a nutrient indicator to reduce calorie intake, either by activating mTOR, by increasing concentrations of cholecystokinin, or moderately decreasing antral pressures (Cota et al. 2006; Blouet and Schwartz 2012; Steinert et al. 2015). Strength training in humans can reduce the intake of fat and calories after 8 months of training (Bales et al. 2012).

This study provides important information for the development of nonpharmacological strategies for the treatment of diabetes mellitus, particularly in diabetic individuals that depend on exogenous insulin. Note that exogenous insulin alone is not sufficient to stimulate MPS in the postprandial state, which requires a greater intake of protein through a diet supplemented with essential amino acids rich in leucine. In combination with regimented resistance training, this strategy helps to maintain and build strong muscles and bones.

## Conclusions

It can be concluded that chronic leucine supplementation and resistance training are effective in improving muscle strength and normalizing fasting plasma glucose and the postprandial phase in diabetic animals. However, resistance training was responsible for major functional adaptations in skeletal muscle of diabetic rats, promoting greater increases in strength and muscle mass compared to leucine supplementation alone. However, the combination of both resistance training and leucine appear to have synergistic effects on MPS, conceivably by regulating the mTOR‐p70S6K pathway.

## Conflicts of Interest

All authors declare no conflict of interest.

## References

[phy213273-bib-0001] Adams, G. R. , and F. Haddad . 1996 The relationships among IGF‐1, DNA content, and protein accumulation during skeletal muscle hypertrophy. J. Appl. Physiol. (1985) 81:2509–2516. 9018499 10.1152/jappl.1996.81.6.2509

[phy213273-bib-0002] Anderson, K. D. , M. Abdul , and O. Steward . 2004 Quantitative assessment of deficits and recovery of forelimb motor function after cervical spinal cord injury in mice. Exp. Neurol. 190:184–191. 15473991 10.1016/j.expneurol.2004.06.029

[phy213273-bib-0003] Baar, K. , and K. Esser . 1999 Phosphorylation of p70S6kcorrelates with increased skeletal muscle mass following resistance exercise. Am. J. Physiol. 276:C120–C127. 9886927 10.1152/ajpcell.1999.276.1.C120

[phy213273-bib-0004] Bales, C. W. , V. H. Hawk , E. O. Granville , S. B. Rose , T. Shields , L. Bateman , et al. 2012 Aerobic and resistance training effects on energy intake: the STRRIDE‐AT/RT study. Med. Sci. Sports Exerc. 44:2033–2039. 22525775 10.1249/MSS.0b013e318259479a PMC3652977

[phy213273-bib-0005] Blouet, C. , and G. J. Schwartz . 2012 Brainstem nutrient sensing in the nucleus of the solitary tract inhibits feeding. Cell Metab. 16:579–587. 23123165 10.1016/j.cmet.2012.10.003 PMC3537851

[phy213273-bib-0006] Burd, N. A. , J. E. Tang , D. R. Moore , and S. M. Phillips . 2009 Exercise training and protein metabolism: influences of contraction, protein intake, and sex‐based differences. J. Appl. Physiol. 106:1692–1701. 19036897 10.1152/japplphysiol.91351.2008

[phy213273-bib-0007] Burd, N. A. , A. M. Holwerda , K. C. Selby , D. W. West , A. W. Staples , N. E. Cain , et al. 2010 Resistance exercise volume affects myofibrillar protein synthesis and anabolic signalling molecule phosphorylation in young men. J. Physiol. 588:3119–3130. 20581041 10.1113/jphysiol.2010.192856 PMC2956949

[phy213273-bib-0008] Call, J. A. , J. N. Mckeehen , S. A. Novotny , and D. A. Lowe . 2010 Progressive resistance voluntary wheel running in the mdx mouse. Muscle Nerve 42:1517–1524. 10.1002/mus.21764 PMC3392646 21104862

[phy213273-bib-0009] Cartee, G. D. 2015 Mechanisms for greater insulin‐stimulated glucose uptake in normal and insulin resistant skeletal muscle after acute exercise. Am. J. Physiol. Endocrinol. Metab. 309:E949–E959. 26487009 10.1152/ajpendo.00416.2015 PMC4816200

[phy213273-bib-0010] Castaneda, C. , J. E. Layne , L. Munoz‐orians , P. L. Gordon , J. Walsmith , M. Foldvari , et al. 2002 A randomized controlled trial of resistance exercise training to improve glycemic control in older adults with type 2 diabetes. Diabetes Care 25:2335–2341. 12453982 10.2337/diacare.25.12.2335

[phy213273-bib-0011] Chen, W. C. , W. C. Huang , C. C. Chiu , Y. K. Chang , and C. C. Huang . 2014 Whey protein improves exercise performance and biochemical profiles in trained mice. Med. Sci. Sports Exerc. 46:1517–1524. 24504433 10.1249/MSS.0000000000000272 PMC4186725

[phy213273-bib-0012] Cota, D. , K. Proulx , K. A. Smith , S. C. Kozma , G. Thomas , S. C. Woods , et al. 2006 Hypothalamic mTOR signaling regulates food intake. Science 312:927–930. 16690869 10.1126/science.1124147

[phy213273-bib-0013] Dreyer, H. C. , S. Fujita , J. Cadenas , D. L. Chinked , E. Volpi , and B. B. Rasmussen . 2006 Resistance exercise increases AMPK activity and reduces 4E‐BP1 phosphorylation and protein synthesis in human skeletal muscle. J. Physiol. 576:613–624. 16873412 10.1113/jphysiol.2006.113175 PMC1890364

[phy213273-bib-0014] Dreyer, H. C. , M. J. Drummond , B. Pennings , S. Fujita , E. L. Glynn , D. L. Chinkes , et al. 2008 Leucine‐enriched essential amino acid and carbohydrate ingestion following resistance exercise enhances mTOR signaling and protein synthesis in human muscle. Am. J. Physiol. Endocrinol. Metab. 294:E392–E400. 18056791 10.1152/ajpendo.00582.2007 PMC2706121

[phy213273-bib-0015] D'Souza, D. M. , D. Al‐sajee , and T. J. Hawke . 2013 Diabetic myopathy: impact of diabetes mellitus on skeletal muscle progenitor cells. Front. Physiol. 4:1–7. 24391596 10.3389/fphys.2013.00379 PMC3868943

[phy213273-bib-0016] Frontera, W. R. , and J. Ochala . 2015 Skeletal muscle: a brief review of structure and function. Calcif. Tissue Int. 96:183–195. 25294644 10.1007/s00223-014-9915-y

[phy213273-bib-0017] Gao, Z. , R. A. Young , G. Li , H. Najafi , C. Buettger , S. S. Sukumvanich , et al. 2003 Distinguishing features of leucine and alpha‐ketoisocaproate sensing in pancreatic beta‐cells. Endocrinology 144:1949–1957. 12697702 10.1210/en.2002-0072

[phy213273-bib-0018] Goodman, C. A. , D. L. Mayhew , and T. A. Hornberger . 2011 Recent progress toward understanding the molecular mechanisms that regulate skeletal muscle mass. Cell. Signal. 23:1896–1906. 21821120 10.1016/j.cellsig.2011.07.013 PMC3744211

[phy213273-bib-0019] Guimarães‐ferreira, L. , J. M. Cholewa , M. A. Naimo , X. I. Zhi , D. Magagnin , R. B. de Sá , et al. 2014 Synergistic effects of resistance training and protein intake: practical aspects. Nutrition 30:1097–1103. 24751198 10.1016/j.nut.2013.12.017

[phy213273-bib-0020] Gumucio, J. P. , and C. L. Mendias . 2013 Atrogin‐1, MuRF‐1, and sarcopenia. Endocrine 43:12–21. 22815045 10.1007/s12020-012-9751-7 PMC3586538

[phy213273-bib-0021] Haddad, F. , and G. R. Adams . 2002 Selected contribution: acute cellular and molecular responses to resistance exercise. J. Appl. Physiol. 93:394–403. 12070230 10.1152/japplphysiol.01153.2001

[phy213273-bib-0022] Ham, D. J. , M. K. Caldow , G. S. Lynch , and R. Koopman . 2014 Leucine as a treatment for muscle wasting: a critical review. Clin. Nutr. 33:937–945. 25444557 10.1016/j.clnu.2014.09.016

[phy213273-bib-0023] Holloszy, J. O. 2005 Exercise‐induced increase in muscle insulin sensitivity. J. Appl. Physiol. 99:338–343. 16036907 10.1152/japplphysiol.00123.2005

[phy213273-bib-0024] Holten, M. K. , M. Zacho , M. Gaster , C. Juel , J. F. Wojtaszewski , and F. Dela . 2004 Strength training increases insulin‐mediated glucose uptake, GLUT4 content, and insulin signaling in skeletal muscle in patients with type 2 diabetes. Diabetes 53:294–304. 14747278 10.2337/diabetes.53.2.294

[phy213273-bib-0025] Hornberger, T. A. Jr , and R. P. Farrar . 2004 Physiological hypertrophy of the FHL muscle following 8 weeks of progressive resistance exercise in the rat. Can. J. Appl. Physiol. 29:16–31. 15001801 10.1139/h04-002

[phy213273-bib-0026] Huang, H. H. , K. Farmer , J. Windscheffel , K. Yost , M. Power , D. E. Wright , et al. 2011 Exercise increases insulin content and basal secretion in pancreatic islets in type 1 diabetic mice. Exp. Diabetes Res. 2011:481427. 21912535 10.1155/2011/481427 PMC3170797

[phy213273-bib-0027] Hurley, B. F. , and S. M. Roth . 2000 Strength training in the elderly: effects on risk factors for age‐related diseases. Sports Med. 30:249–268. 11048773 10.2165/00007256-200030040-00002

[phy213273-bib-0029] Kelleher, A. R. , S. R. Kimball , M. D. Dennis , R. J. Schilder , and L. S. Jefferson . 2013 The mTORC1 signaling repressors REDD1/2 are rapidly induced and activation of p70S6K1 by leucine is defective in skeletal muscle of an immobilized rat hindlimb. Am. J. Physiol. Endocrinol. Metab. 304:E229–E236. 23193052 10.1152/ajpendo.00409.2012 PMC3543567

[phy213273-bib-0030] Kennel, P. F. , P. Fonteneau , E. Martin , J. M. Schmidt , M. Azzouz , B. Jacques , et al. 1996 Electromyographical and motor performance studies in the pmn mouse model of neurodegenerative disease. Neurobiol. Dis. 3:137–147. 9173921 10.1006/nbdi.1996.0014

[phy213273-bib-0031] Kim, J. Y. , M. J. Choi , B. So , H. J. Kim , J. K. Seong , and W. Song . 2015 The preventive effects of 8 weeks of resistance training on glucose tolerance and muscle fiber type composition in zucker rats. Diabetes Metab. J. 39:424–433. 26566500 10.4093/dmj.2015.39.5.424 PMC4641972

[phy213273-bib-0032] Krause, M. P. , D. Al‐sajee , D. M. D'Souza , I. A. Rebalka , J. Moradi , M. C. Riddell , et al. 2013 Impaired macrophage and satellite cell infiltration occurs in a muscle‐specific following injury in diabetic skeletal muscle. PLoS ONE 8:e70971. 23951058 10.1371/journal.pone.0070971 PMC3741394

[phy213273-bib-0033] Kubica, N. , D. R. Bolster , P. A. Farrell , S. R. Kimball , and L. S. Jefferson . 2005 Resistance exercise increases muscle protein synthesis and translation of eukaryotic initiation factor 2Bϵ mRNA in a mammalian target of rapamycin‐dependent manner. J. Biol. Chem. 280:7570–7580. 15591312 10.1074/jbc.M413732200

[phy213273-bib-0034] Manders, R. J. , J. P. Little , S. C. Forbes , and D. G. Candow . 2012 Insulinotropic and muscle protein synthetic effects of branched‐chain amino acids: potential therapy for type 2 diabetes and sarcopenia. Nutrients 4:1664–1678. 23201839 10.3390/nu4111664 PMC3509512

[phy213273-bib-0035] Nalbandian, A. , C. Nguyen , V. Katheria , K. J. Llewellyn , M. Badadani , V. Caiozzo , et al. 2013 Exercise training reverses skeletal muscle atrophy in an experimental model of VCP disease. PLoS ONE 8:e76187. 24130765 10.1371/journal.pone.0076187 PMC3794032

[phy213273-bib-0036] Norton, L. E. , and D. K. Layman . 2006 Leucine regulates translation initiation of protein synthesis in skeletal muscle after exercise. J. Nutr. 136:533S–537S. 16424142 10.1093/jn/136.2.533S

[phy213273-bib-0037] Ogasawara, R. , K. Kobayashi , A. Tsutaki , K. Lee , T. Abe , S. Fujita , et al. 2013 mTOR signaling response to resistance exercise is altered by chronic resistance training and detraining in skeletal muscle. J. Appl. Physiol. 114:934–940. 23372143 10.1152/japplphysiol.01161.2012

[phy213273-bib-0038] Oliveira, C. A. , M. F. Paiva , C. A. Mota , C. Ribeiro , J. A. Leme , E. Luciano , et al. 2010 Exercise at anaerobic threshold intensity and insulin secretion by isolated pancreatic islets of rats. Islets 2:240–246. 21099318 10.4161/isl.2.4.12266 PMC3322538

[phy213273-bib-0039] Pasiakos, S. M. , H. L. Mcclung , J. P. Mcclung , L. M. Margolis , N. E. Andersen , G. J. Cloutier , et al. 2011 Leucine‐enriched essential amino acid supplementation during moderate steady state exercise enhances post exercise muscle protein synthesis. Am. J. Clin. Nutr. 94:809–818. 21775557 10.3945/ajcn.111.017061

[phy213273-bib-0028] Pedroso, J. A. , T. T. Zampieri , and J. Jr Donato . 2015 Reviewing the Effects of L‐Leucine Supplementation in the Regulation of Food Intake, Energy Balance, and Glucose Homeostasis. Nutrients 7:3914–3937. 26007339 10.3390/nu7053914 PMC4446786

[phy213273-bib-0040] Phillips, S. M. 2014 A brief review of critical processes in exercise‐induced muscular hypertrophy. Sports Med. 44:S71–S77. 24791918 10.1007/s40279-014-0152-3 PMC4008813

[phy213273-bib-0041] Phillips, S. M. , K. D. Tipton , A. Aarsland , S. E. Wolf , and R. R. Wolfe . 1997 Mixed muscle protein synthesis and breakdown after resistance exercise in humans. Am. J. Physiol. 273:E99–E107. 9252485 10.1152/ajpendo.1997.273.1.E99

[phy213273-bib-0042] Reeves, P. G. , F. H. Nielsen , and G. C. Jr Fahey . 1993 AIN‐93 purified diets for laboratory rodents: final report of the American Institute of Nutrition ad hoc writing committee on the reformulation of the AIN‐76A rodent diet. J. Nutr. 123:1939–1951. 8229312 10.1093/jn/123.11.1939

[phy213273-bib-0043] Rieu, I. , C. Sornet , G. Bayle , J. Prugnaud , C. Pouyet , M. Balage , et al. 2003 Leucine‐supplemented meal feeding for ten days beneficially affects postprandial muscle protein synthesis in old rats. J. Nutr. 133:1198–1205. 12672943 10.1093/jn/133.4.1198

[phy213273-bib-0044] Smith, T. J. , and C. A. Stanley . 2008 Untangling the glutamate dehydrogenase allosteric nightmare. Trends Biochem. Sci. 33:557–564. 18819805 10.1016/j.tibs.2008.07.007

[phy213273-bib-0045] Spiering, B. A. , W. J. Kraemer , J. M. Anderson , L. E. Armstrong , B. C. Nindl , J. S. Volek , et al. 2008 Resistance exercise biology: manipulation of resistance exercise programme variables determines the responses of cellular and molecular signalling pathways. Sports Med. 38:527–540. 18557656 10.2165/00007256-200838070-00001

[phy213273-bib-0046] Srikanthan, P. , and A. S. Karlamangla . 2014 Muscle mass index as a predictor of longevity in older adults. Am. J. Med. 127:547–553. 24561114 10.1016/j.amjmed.2014.02.007 PMC4035379

[phy213273-bib-0047] Steinert, R. E. , M. F. Landrock , S. S. Ullrich , S. Standfield , B. Otto , M. Horowitz , et al. 2015 Effects of intraduodenal infusion of the branched‐chain amino acid leucine on ad libitum eating, gut motor and hormone functions, and glycemia in healthy men. Am. J. Clin. Nutr. 102:820–827. 26289436 10.3945/ajcn.115.114488

[phy213273-bib-0048] Takala, T. O. , P. Nuutila , J. Knuuti , M. Luotolahti , and H. Yki‐jarvinen . 1999 Insulin action on heart and skeletal muscle glucose uptake in weight lifters and endurance athletes. Am. J. Physiol. 276:E706–E711. 10198307 10.1152/ajpendo.1999.276.4.E706

[phy213273-bib-0049] Tsubuku, S. , K. Hatayama , T. Katsumata , N. Nishimura , K. Mawatari , M. Smriga , et al. 2004 Thirteen‐week oral toxicity study of branched‐chain amino acids in rats. Int. J. Toxicol. 23:119–126. 15204732 10.1080/10915810490444424

[phy213273-bib-0050] Vianna, D. , G. F. Resende , F. L. Torres‐Leal , L. C. Pantaleão , J. Jr Donato , and J. Tirapegui . 2012 Long‐term leucine supplementation reduces fat mass gain without changing body protein status of aging rats. Nutrition 28:182–189. 21872432 10.1016/j.nut.2011.04.004

[phy213273-bib-0051] Wilkinson, D. J. , T. Hossain , D. S. Hill , B. E. Phillips , H. Crossland , J. Williams , et al. 2013 Effects of leucine and its metabolite *β*‐hydroxy‐*β*‐methylbutyrate on human skeletal muscle protein metabolism. J. Physiol. 591:2911–2923. 23551944 10.1113/jphysiol.2013.253203 PMC3690694

[phy213273-bib-0052] Workeneh, B. , and M. Bajaj . 2013 The regulation of muscle protein turnover in diabetes. Int. J. Biochem. Cell Biol. 45:2239–2244. 23838169 10.1016/j.biocel.2013.06.028

[phy213273-bib-0053] Zanchi, E. Z. , L. Guimarães‐ferreira , M. A. Siqueira‐filho , V. Felitti , H. Nicastro , C. Jr Bueno , et al. 2012 Dose and latency effects of leucine supplementation in modulating glucose homeostasis: opposite effects in healthy and glucocorticoid‐induced insulin‐resistance states. Nutrients 4:1851–1867. 23363994 10.3390/nu4121851 PMC3546611

